# An Abstract Contract Theory for Programs with Procedures

**DOI:** 10.1007/978-3-030-71500-7_8

**Published:** 2021-02-24

**Authors:** Christian Lidström, Dilian Gurov

**Affiliations:** 8 Universidad Autónoma de Madrid, Madrid, Spain; 9 University of Twente, Enschede, The Netherlands; KTH Royal Institute of Technology, Stockholm, Sweden

## Abstract

When developing complex software and systems, *contracts* provide a means for controlling the complexity by dividing the responsibilities among the components of the system in a hierarchical fashion. In specific application areas, dedicated *contract theories* formalise the notion of contract and the operations on contracts in a manner that supports best the development of systems in that area. At the other end, *contract meta-theories* attempt to provide a systematic view on the various contract theories by axiomatising their desired properties. However, there exists a noticeable gap between the most well-known contract meta-theory of Benveniste et al. [5], which focuses on the design of embedded and cyber-physical systems, and the established way of using contracts when developing general software, following Meyer’s design-by-contract methodology [18]. At the core of this gap appears to be the notion of *procedure*: while it is a central unit of composition in software development, the meta-theory does not suggest an obvious way of treating procedures as components.

In this paper, we provide a first step towards a contract theory that takes procedures as the basic building block, and is at the same time an instantiation of the meta-theory. To this end, we propose an abstract contract theory for sequential programming languages with procedures, based on *denotational semantics*. We show that, on the one hand, the specification of contracts of procedures in *Hoare logic*, and their procedure-modular verification, can be cast naturally in the framework of our abstract contract theory. On the other hand, we also show our contract theory to fulfil the axioms of the meta-theory. In this way, we give further evidence for the utility of the meta-theory, and prepare the ground for combining our instantiation with other, already existing instantiations.

## 1 Introduction

*Contracts.* Loosely speaking, a *contract* for a software or system component is a means of specifying that the component obliges itself to guarantee a certain behaviour or result, provided that the user (or client) of the component obliges itself to fulfil certain constraints on how it interacts with the component.

* This work has been funded by the Swedish Governmental Agency for Innovation Systems (VINNOVA) under the AVerT project 2018-02727.

One of the earliest inspirations for the notion of software contracts came from the works of Floyd [10] and Hoare [15]. One outcome of this was *Hoare logic*, which is a way of assigning meaning to sequential programs *axiomatically*, through so-called Hoare triples. A Hoare triple $\{P\}S\{Q\}$ consists of two assertions $P$ and $Q$ over the program variables, called the pre-condition and post-condition, respectively, and a program $S$. The triple states that if the pre-condition $P$ holds prior to executing $S$, then, if execution of $S$ terminates, the post-condition $Q$ will hold upon termination. With the help of additional, so-called *logical variables*, one can specify, with a Hoare triple, the desired relationship between the final values of certain variables (such as the return value of a procedure) and the initial values of certain other variables (such as the formal parameters of the procedure).

This style of specifying contracts has been advocated by Meyer [18], together with the design methodology Design-by-Contract. A central characteristic of this methodology is that it is well-suited for *independent implementation and verification*, where software components are developed independently from each other, based solely on the contracts, and without any knowledge of the implementation details of the other components.

*Contract Theories.* Since then, many other contract theories have emerged, such as Rely/Guarantee reasoning [16,22] and a number of Assume/Guarantee contract theories [4,6]. A contract theory typically formalises the notion of contract, and develops a number of operations on contracts that support typical design steps. This in turn has lead to a few developments of contract *meta-theories* (e.g. [5,2,8]), which aim at unifying these, in many cases incompatible, contract theories. The most comprehensive, and well-known, of these, is presented in Benveniste et al. [5], and is concerned specifically with the design of cyber-physical systems. Here, all properties are derived from a most abstract notion of a contract. The meta-theory focuses on the notion of contract *refinement*, and the operations of contract *conjunction* and *composition*. The intention behind refinement and composition is to support a top-down design flow, where contracts are decomposed iteratively into sub-contracts; the task is then to show that the composition of the sub-contracts refines the original contract. These operations are meant to enable *independent development* and *reuse* of components. In addition, the operation of conjunction is intended to allow the superimposition of contracts over *the same* component, when they concern different aspects of its behaviour. This also enables *component reuse*, by allowing contracts to reveal only the behaviour relevant to the different use cases.

*Motivation and Contribution.* The meta-theory of Benveniste et al. focuses on the design of embedded and cyber-physical systems. However, there exists a noticeable gap between this meta-theory and the way contracts are used when developing general software following Meyer's design-by-contract methodology. At the core of this gap appears to be the notion of *procedure*[1]. While the proce-

[1] We use the term "procedure", rather than "function" or "method", to refer to the well-known control abstraction mechanism of imperative programming languages.

dure is a central unit of composition in software development, the meta-theory does not suggest an obvious way of treating procedures as components. This situation is not fully satisfactory, since the software components of most embedded systems are implemented with the help of procedures (a typical C-module, for instance, would consist of a main function and a number of *helper* functions), and their development should ideally follow the same design flow as that of the embedded system as a whole.

In this paper we provide a first step towards a contract theory that takes procedures as the basic building block, and at the same time respects the axioms of the meta-theory. Our contract theory is abstract, so that it can be instantiated to any procedural language, and similarly to the meta-theory, is presented at the semantics level only. Then, in the context of a simplistic imperative programming language with procedures and its denotational semantics, we show that the specification of contracts of procedures in *Hoare logic*, and their procedure-modular verification, can be cast in the framework of our abstract contract theory. We also show that our contract theory is an instance of the meta-theory of Benveniste et al. With this we expect to contribute to the bridging of the gap mentioned above, and to give a formal justification of the design methodology supported by the meta-theory, when applied to the software components of embedded systems. Several existing contract theories have already been shown to instantiate the meta-theory. In providing a contract theory for procedural programs that also instantiates it, we increase the value of the meta-theory by providing further evidence for its universality. In addition, we prepare the theoretical ground for combining our instantiation with other instantiations, which may target components not to be implemented in software.

Our theoretical development should be seen as a proof-of-concept. In future work it will need to be extended to cover more programming language features, such as object orientation, multi-threading, and exceptions.

*Related Work.* Software contracts and operations on contracts have long been an area of intensive research, as evidenced, e.g., by [1]. We briefly mention some works related to our theory, in addition to the already mentioned ones.

Reasoning from multiple Hoare triples is studied in [21], in the context of unavailable source code, where new properties cannot be derived by re-verification. In particular, it is found that two Hoare-style rules, the standard rule of consequence and a generalised normalisation rule, are sufficient to infer, from a set of existing contracts for a procedure, any contract that is semantically entailed.

Often-changing source code is a problem for contract-based reasoning and contract reuse. In [13], abstract method calls are introduced to alleviate this problem. Fully abstract contracts are then introduced in [7], allowing reasoning about software to be decoupled from contract applicability checks, in a way that not all verification effort is invalidated by changes in a specification.

The relation between behavioural specifications and assume/guarantee-style contracts for modal transition systems is studied in [2], which shows how to build a contract framework from any specification theory supporting composition and refinement. This work is built on in [9], where a formal contract framework based on temporal logic is presented, allowing verification of correctness of contract refinement relative to a specific decomposition.

A survey of behavioural specification languages [14] found that existing languages are well-suited for expressing properties of software components, but it is a challenge to express how components interact, making it difficult to reason about system and architectural level properties from detailed design specifications. This provides additional evidence for the gap between contracts used in software verification and contracts as used in system design.

*Structure.* The paper is organised as follows. Section 2 recalls the concept of contract based design and the contract meta-theory considered in the present paper. In Section 3 we present a denotational semantics for programs with procedures, including a semantics for contracts for use in procedure-modular verification. Next, Section 4 presents our abstract contract theory for sequential programs with procedures. Then, we show in Section 5 that our contract theory fulfils the axioms of the meta-theory, while in Section 6 we show how the specification of contracts of procedures in Hoare logic and their procedure-modular verification can be cast in the framework of our abstract contract theory. We conclude with Section 7.

## 2 Contract Based Design

This section describes the concept of *contract based design*, and motivates its use in cyber-physical systems development. We then recall the contract meta-theory by Benveniste et al. [5].

### 2.1 Contract Based Design of Cyber-Physical Systems

*Contract based design* is an approach to systems design, where the system is developed in a top-down manner through the use of contracts for components, which are incrementally assembled so that they preserve the desired system-wide properties. Contracts are typically described by a set of *assumptions* the component makes on its environment, and a set of *guarantees* on the component's behaviour, given that it operates in an environment adhering to the assumptions [5].

Present-day cyber-physical systems, such as those found in the automotive, avionics and other industries, are extremely complex. Products assembled by Original Equipment Manufacturers (OEMs) often consist of components from a number of different suppliers, all using their own specialised design processes, system architectures, development platforms, and tools. This is also true inside the OEMs, where there are different teams with different viewpoints of the system, and their own design processes and tools. In addition, the system itself has several different aspects that need to be managed, such as the architecture, safety and security requirements, functional behaviour, and so on. Thus, a rigorous design framework is called for that can solve these design-chain management issues.

Contract based design addresses these challenges through the principles, at the specification level, of *refinement* and *abstraction*, which are processes for managing the design flow between different layers of abstraction, and *composition* and *decomposition*, which manage the flow at the same level of abstraction. Generally, when designing a system, at the top level of abstraction there will be an overall system specification (or contract). This *top-level contract* is then refined, to provide a more concrete contract for the system, and decomposed, in order to obtain contracts for the sub-systems, and to separate the different viewpoints of the system. A system design typically iterates the decomposition-and-refinement process, resulting in several layers of abstraction, until contracts are obtained that can be directly implemented, or for which implementations already exist. An important requirement on this methodology of hierarchical decomposition and refinement of contracts is that it must guarantee that when the low-level components implement their concrete contracts, and are combined to form the overall system, then the top-level, abstract, contract shall hold.

Furthermore, a contract framework in particular needs to support *independent development* and *component reuse*. That is, specifications for components, and their operations, must allow for components and specifications to be independently designed and implemented, and to be used in different parts of the system, each with their own assumptions on how the other components, the environment, behave. This is achieved through the principle operations on contracts: *refinement*, *composition*, and *conjunction*.

Refinement allows one to extract a contract at the appropriate level of abstraction. A desired property of refinement is that components which have been designed with reference to the more abstract (i.e., weaker) contract do not need to be re-designed after the refinement step. That is, in the early stages of development an OEM may have provided a weak contract for some subsystem to an external supplier, which implemented a component relying on this contract. As development of the system progresses, and the contract is refined, the component supplied externally should still operate according to its guarantees without needing to be changed, when instead assuming the new, refined, contract.

Composition enables one to combine contracts of different components into a contract for the larger subsystem obtained when combining the components. Again, a desirable property is that other components relying on one or more of the individual contracts, can, after composition of the contracts, assume the new contract and still perform its guarantees, without being re-designed, thus ensuring that subsystems can be independently implemented.

Finally, contract conjunction is another way of combining contracts, but now for the different viewpoints of a single component. This allows one to separate a contract into several different, finer contracts for the same component, revealing just enough information for each particular system that depends on it, so that it can be reused in different parts of the system, or in entirely different systems.

## 2.2 A Contract Meta-Theory

We consider the meta-theory described in [5]. The stated purpose of the meta-theory has been to distil the notion of a contract to its essence, so that it can be used in system design methodologies without ambiguities. In particular, the meta-theory has been developed to give support for design-chain management, and to allow *component reuse* and *independent development.* It has been shown that a number of concrete contract theories instantiate it, including assume/guarantee-contracts, synchronous Moore interfaces, and interface theories. To our knowledge, this is the only meta-theory of its purpose and scope.

We now present the formal definitions of the concepts defined in the meta-theory, and the properties that they entail. The meta-theory is defined only in terms of semantics, and it is up to particular concrete instantiations to provide a syntax.

*Components.* The most basic concept in the meta-theory is that of a *component*, which represents any concrete part of the system. Thus, we have an abstract component universe $\mathbb{M}$ with components $m \in \mathbb{M}$. Over pairs of components, we have a *composition* operation $\times$. This operation is partially defined, and two components $m_1$ and $m_2$ are called *composable* when $m_1 \times m_2$ is defined. In such cases, we call $m_1$ an *environment* for $m_2$, and vice versa. In addition, component composition must be both commutative and associative, in order to ensure that different components can be combined in any order.

Typically, components are *open*, in the sense that they contain functionality provided by other components, i.e., their environment. The environment in which a component is to be placed is often unknown at development time, and although a component cannot restrict it, it is designed for a certain context.

*Contracts.* In the meta-theory, the notion of *contract* is defined in terms of sets of components. The contract universe $\mathbb{C} \stackrel{\mathsf{def}}{=} 2^{\mathbb{M}} \times 2^{\mathbb{M}}$ consists of contracts $\mathcal{C} = (E, M)$, where $E$ and $M$ are the sets of *environments* and *implementations* of $\mathcal{C}$, respectively. Importantly, each pair $(m_1, m_2) \in E \times M$ must be composable. This definition is intentionally abstract. The intuition is that contracts separate the responsibilities of a component from the expectations on its environment. Moreover, contracts are best seen as *weak specifications* of components: they should expose just enough information to be adequate for their purpose.

For a component $m$ and a contract $\mathcal{C} = (E, M)$, we shall sometimes write $m \models^E \mathcal{C}$ for $m \in E$, and $m \models^M \mathcal{C}$ for $m \in M$. A contract $\mathcal{C}$ is said to be *consistent* if it has at least one implementation, and *compatible* if it has at least one environment.

*Contract refinement.* For two contracts $\mathcal{C}_1 = (E_1, M_1)$ and $\mathcal{C}_2 = (E_2, M_2)$, $\mathcal{C}_1$ is said to *refine* $\mathcal{C}_2$, denoted $\mathcal{C}_1 \preceq \mathcal{C}_2$, iff $M_1 \subseteq M_2$ and $E_2 \subseteq E_1$. As an axiom of the meta-theory, it is required that the greatest lower bound with respect to refinement exists, for all subsets of $\mathbb{C}$. Table 1 summarises the important properties of refinement and the other operations on contracts that a concrete

**Table 1.** Properties that hold in theories that adhere to the meta-theory.

| # | **Property** |
|---|---|
| 1 | *Refinement.* When $\mathcal{C}_1 \preceq \mathcal{C}_2$, every implementation of $\mathcal{C}_1$ is also an implementation of $\mathcal{C}_2$. |
| 2 | *Shared refinement.* Any contract refining $\mathcal{C}_1 \wedge \mathcal{C}_2$ also refines $\mathcal{C}_1$ and $\mathcal{C}_2$. Any implementation of $\mathcal{C}_1 \wedge \mathcal{C}_2$ is a shared implementation of $\mathcal{C}_1$ and $\mathcal{C}_2$. Any environment for $\mathcal{C}_1$ and $\mathcal{C}_2$ is an environment for $\mathcal{C}_1 \wedge \mathcal{C}_2$. |
| 3 | *Independent implementability.* Compatible contracts can be independently implemented. |
| 4 | *Independent refinement.* For all contracts $\mathcal{C}_i$ and $\mathcal{C}'_i, i \in I$, if $\mathcal{C}_i, i \in I$ are compatible and $\mathcal{C}'_i \preceq \mathcal{C}_i, i \in I$ hold, then $\mathcal{C}'_i, i \in I$ are compatible and $\bigotimes_{i \in I} \mathcal{C}'_i \preceq \bigotimes_{i \in I} \mathcal{C}_i$ |
| 5 | *Commutativity, sub-associativity.* For any finite sets of contracts $\mathcal{C}_i, i = 1, \ldots, n$, $\mathcal{C}_1 \otimes \mathcal{C}_2 = \mathcal{C}_2 \otimes \mathcal{C}_1$ and $\bigotimes_{1 \leq i \leq n} \mathcal{C}_i \preceq (\bigotimes_{1 \leq i < n} \mathcal{C}_i) \otimes \mathcal{C}_n$ holds. |
| 6 | *Sub-distributivity.* The following holds, if all contract compositions in the formula are well defined: $((\mathcal{C}_{11} \wedge \mathcal{C}_{21}) \otimes (\mathcal{C}_{12} \wedge \mathcal{C}_{22})) \preceq ((\mathcal{C}_{11} \otimes \mathcal{C}_{12}) \wedge (\mathcal{C}_{21} \otimes \mathcal{C}_{22}))$ |

contract theory needs to possess in order to be considered an instance of the meta-theory.

*Contract conjunction.* The *conjunction* of two contracts $\mathcal{C}_1$ and $\mathcal{C}_2$, denoted $\mathcal{C}_1 \wedge \mathcal{C}_2$, is defined as their greatest lower bound w.r.t. the refinement order. (The intention is that $(E_1, M_1) \wedge (E_2, M_2)$ should equal $(E_1 \cup E_2, M_1 \cap M_2)$; however, this cannot be taken as the definition since not every such pair necessarily constitutes a contract.) Then, we have the three desirable properties of conjunction listed in Table 1, which together are referred to as *shared refinement*.

*Contract composition.* The *composition* of two contracts $\mathcal{C}_1 = (E_1, M_1)$ and $\mathcal{C}_2 = (E_2, M_2)$, denoted $\mathcal{C}_1 \otimes \mathcal{C}_2 = (E, M)$, is defined when every two components $m_1 \in M_1$ and $m_2 \in M_2$ are composable, and must then be the least contract, w.r.t. the refinement order, satisfying the following conditions:

(i) $m_1 \in M_1 \wedge m_2 \in M_2 \Rightarrow m_1 \times m_2 \in M$;
(ii) $e \in E \wedge m_1 \in M_1 \Rightarrow m_1 \times e \in E_2$; and
(iii) $e \in E \wedge m_2 \in M_2 \Rightarrow e \times m_2 \in E_2$.

If all of the above is satisfied, then properties 3-6 of Table 1 hold. The intention is that composing two components implementing $\mathcal{C}_1$ and $\mathcal{C}_2$ should yield an implementation of $\mathcal{C}_1 \otimes \mathcal{C}_2$, and composing an environment of $\mathcal{C}_1 \otimes \mathcal{C}_2$ with an implementation of $\mathcal{C}_1$ should result in a valid environment for $\mathcal{C}_2$, and vice versa. This is important in order to enable independent development.

## 3 Denotational Semantics of Programs and Contracts

In this section we summarise the background needed to understand the formal developments later in the paper. First, we recall the standard denotational semantics of programs with procedures on a typical toy programming language.

Next, we summarise Hoare logic and contracts, and provide a semantic justification of procedure-modular verification, also based on denotational semantics.

### 3.1 The Denotational Semantics of Programs with Procedures

This section sketches the standard presentation of denotational semantics for procedural languages, as presented in textbooks such as [23,19]. This semantics is the inspiration for the definition of components in our abstract contract theory in Section 4.1. We start with a simplistic programming language not involving procedures, and add procedures later to the language.

The following toy sequential programming language is typically used to present the denotational semantics of imperative languages:

$$S \ ::= \ \mathsf{skip} \mid \mathsf{x} := \mathsf{a} \mid S_1; S_2 \mid \mathsf{if}\ b\ \mathsf{then}\ S_1\ \mathsf{else}\ S_2 \mid \mathsf{while}\ b\ \mathsf{do}\ S$$

where $S$ ranges over statements, $a$ over arithmetic expressions, and $b$ over Boolean expressions.

To define the denotational semantics of the language, we define the set **State** of program states. A state $s \in \mathbf{State}$ is a mapping from the program variables to, for simplicity, the set of integers.

The *denotation* of a statement $S$, denoted $[\![S]\!]$, is typically given as a partial function $\mathbf{State} \hookrightarrow \mathbf{State}$ such that $[\![S]\!]\,(s) = s'$ whenever executing statement $S$ from the initial state $s$ terminates in state $s'$. In case that executing $S$ from $s$ does not terminate, the value of $[\![S]\!]\,(s)$ is undefined. The definition of $[\![S]\!]$ proceeds by induction on the structure of $S$. For example, the meaning of sequential composition of statements is usually captured with relation composition, as given by the equation $[\![S_1; S_2]\!] \stackrel{\mathsf{def}}{=} [\![S_1]\!] \circ [\![S_2]\!]$. For the treatment of the remaining statements of the language, the reader is referred to [23,19].

The definition of denotation captures through its type (as a partial function) that the execution of statements is deterministic. For non-deterministic programs, the type of denotations is relaxed to $[\![S]\!] \subseteq \mathbf{State} \times \mathbf{State}$; then, $(s, s') \in [\![S]\!]$ captures that there is an execution of $S$ starting in $s$ that terminates in $s'$. For technical reasons that will become clear below, we shall use this latter denotation type in our treatment.

Note that we could alternatively have chosen $\mathbf{State}^+$ as the denotational domain, and most results would still hold in the context of finite-trace semantics. However, we chose to develop the theory with a focus on Hoare-logic and deductive verification. In fact, the domain $\mathbf{State} \times \mathbf{State}$ can be seen as a special case of finite traces. In future work, we will also investigate concrete contract languages based on this semantics, and extend the theory for that context.

*Procedures and Procedure Calls.* To extend the language and its denotational semantics with procedures and procedure calls, we follow again the approach of [23], but adapt it to an "open" setting, where some called procedures might not be declared. We consider programs in the context of a finite set $\mathcal{P}$ of procedure names (of some larger, "closed" program), and a set of *procedure declarations* of the form $\mathsf{proc}\ p\ \mathsf{is}\ S_p$, where $p \in \mathcal{P}$. Further, we extend the toy programming language with the statement $\mathsf{call}\ p$.

**Listing 1.1.** An even-odd toy program.

```
proc even is if n = 0 then r := 1 else (n := n − 1; call odd);
proc odd  is if n = 0 then r := 0 else (n := n − 1; call even)
```

As an example, Listing 1.1 shows a (closed) program in the toy language, implementing two mutually recursive procedures. The procedures check whether the value of the global variable $n$ is even or odd, respectively, and assign the corresponding truth value to the variable $r$.

Due to the (potential) recursion in the procedure declarations, the denotation of $\mathsf{call}\ p$, and thus of the whole language, cannot be defined by structural induction as directly as before. We therefore define, for any set $P \subseteq \mathcal{P}$ of procedure names, the set $\mathbf{Env}_P = P \to 2^{\mathbf{State}\times\mathbf{State}}$ of *procedure environments*, each environment $\rho \in \mathbf{Env}_P$ thus providing a denotation for each procedure in $P$.

Let $\mathbf{Env} \stackrel{\mathsf{def}}{=} \bigcup_{P\subseteq\mathcal{P}} \mathbf{Env}_P$ be the set of all procedure environments. We define a partial order relation $\sqsubseteq$ on procedure environments, as follows. For any two procedure environments $\rho \in \mathbf{Env}_P$ and $\rho' \in \mathbf{Env}_{P'}$, $\rho \sqsubseteq \rho'$ if and only if $P \subseteq P'$ and $\forall p \in P.\ \rho(p) \subseteq \rho'(p)$.

Recall that a *complete lattice* is a partial order, every set of elements of which has a greatest lower bound (*glb*) within the domain of the lattice (see, e.g., [23]). It is easy to show that for any $P \subseteq \mathcal{P}$, $(\mathbf{Env}_P, \sqsubseteq)$ is a complete lattice, since a greatest lower bound will exist within $\mathbf{Env}_P$. Then, the least upper bound (*lub*) $\rho_1 \sqcup \rho_2$ of any two function environments $\rho_1 \in \mathbf{Env}_{P_1}$ and $\rho_2 \in \mathbf{Env}_{P_2}$ also exists, and is the environment $\rho \in \mathbf{Env}_{P_1\cup P_2}$ such that $\forall p \in P_1 \cup P_2.\ \rho(p) = \rho_1(p) \cup \rho_2(p)$.

We will sometimes need a procedure environment that maps every procedure in $P$ to $\mathbf{State} \times \mathbf{State}$, and we shall denote this environment by $\rho_P^\top$.

Next, for sets of procedures, we shall need the notion of *interface*, which is a pair $(P^-, P^+)$ of disjoint sets of procedure names, where $P^+ \subseteq \mathcal{P}$ is a set of *provided* (or declared) procedures, and $P^- \subseteq \mathcal{P}$ a set of *required* (or called, but not declared) ones.

Then, we (re)define the notion of denotation of statements $S$ in the context of a given interface $(P^-, P^+)$ and environments $\rho^- \in \mathbf{Env}_{P^-}$ and $\rho^+ \in \mathbf{Env}_{P^+}$, and denote it by $[\![S]\!]_{\rho^-}^{\rho^+}$. In particular, we define $[\![\mathsf{call}\ p]\!]_{\rho^-}^{\rho^+}$ as $\rho^-(p)$ when $p \in P^-$ and as $\rho^+(p)$ when $p \in P^+$.

Intuitively, the denotation of a call to a procedure should be equal to the denotation of the body of the latter. We therefore introduce, given an environment $\rho^- \in \mathbf{Env}_{P^-}$, the function $\xi : \mathbf{Env}_{P^+} \to \mathbf{Env}_{P^+}$ defined by $\xi(\rho^+)(p) \stackrel{\mathsf{def}}{=} [\![S_p]\!]_{\rho^-}^{\rho^+}$ for any $\rho^+ \in \mathbf{Env}_{P^+}$ and $p \in P^+$, and consider its fixed points. By the Knaster-Tarski Fixed-Point Theorem (as stated, e.g., in [23]), since $(\mathbf{Env}_{P^+}, \sqsubseteq)$ is a complete lattice and $\xi$ is monotonic, $\xi$ has a least fixed-point $\rho_0^+$.

Finally, we define the notion of *standard denotation* of statement $S$ in the context of a given interface $(P^-, P^+)$ and environment $\rho^- \in \mathbf{Env}_{P^-}$, denoted $[\![S]\!]_{\rho^-}$, by $[\![S]\!]_{\rho^-} \stackrel{\text{def}}{=} [\![S]\!]_{\rho^-}^{\rho_0^+}$, where $\rho_0^+$ is the least fixed-point defined above.

For example, for the closed program in Listing 1.1, we have an interface with $P^+ = \{even, odd\}$ and $P^- = \varnothing$. Then, $(s, s') \in [\![S_{even}]\!]_{\rho^-}^{\rho^+}$ if either $s(n) = 0$ and $s' = s[r \mapsto 1]$, or else if $s(n) > 0$ and $(s[n \mapsto s(n) - 1], s') \in \rho^+(odd)$. The denotation $[\![S_{odd}]\!]_{\rho^-}^{\rho^+}$ is analogous. The resulting least fixed-point $\rho_0^+$ is such that $(s, s') \in [\![S_{even}]\!]_{\rho^-}$, or equivalently $(s, s') \in [\![S_{even}]\!]_{\rho^-}^{\rho_0^+}$, whenever $s(n) \geq 0$, and either $s(n)$ is even and then $s'(n) = 0$ and $s'(r) = 1$, or else $s(n)$ is odd and then $s'(n) = 0$ and $s'(r) = 0$. The standard denotation $[\![S_{odd}]\!]_{\rho^-}$ of *odd* is analogous.

### 3.2 Hoare Logic and Contracts

In this section we summarise the denotational semantics of Hoare logic and the semantic justification of procedure-modular verification, as developed by the second author in [12]. These formalisations serve as the starting point for the definition of contracts in our contract theory developed in Section 4.2.

*Hoare Logic.* The basic judgement of Hoare logic [15] is the Hoare triple, written $\{P\}S\{Q\}$, where $P$ and $Q$ are assertions over the program state, and $S$ is a program statement. The Hoare triple signifies that if the statement $S$ is executed from a state that satisfies $P$ (called the pre-condition), and if this execution terminates, then the final state of the execution will satisfy $Q$ (called the post-condition). Additionally, so-called *logical variables* can be used within a Hoare triple, to specify the desired relationship between the values of variables after execution and the values of variables before execution. The values of the program variables are defined by the notion of state; to give a meaning to the logical variables we shall use *interpretations* $\mathcal{I}$. We shall write $s \models_{\mathcal{I}} P$ to signify that the assertion $P$ is true w.r.t. state $s$ and interpretation $\mathcal{I}$. The formal validity of a Hoare triple is denoted by $\models_{par} \{P\}S\{Q\}$, where the subscript signifies that validity is in terms of *partial correctness*, where termination of the execution of $S$ is not required.

An example of a Hoare triple, stating the desired behaviour of procedure *odd* from Listing 1.1, is shown below, where we use the logical variable $n_0$ to capture to the value of $n$ prior to execution of *odd*:

$$\{n \geq 0 \wedge n = n_0\}\ S_{odd}\ \{(n_0 \bmod 2 = 0 \Rightarrow r = 0) \wedge (n_0 \bmod 2 = 1 \Rightarrow r = 1)\} \quad (1)$$

Procedure *even* is specified analogously.

Hoare logic comes with a proof calculus for reasoning in terms of Hoare triples, consisting of proof rules for the different types of statements of the programming language. An example is the rule for sequential composition:

$$\frac{\{P\}\ S_1\ \{R\} \qquad \{R\}\ S_2\ \{Q\}}{\{P\}\ S_1; S_2\ \{Q\}}\ \textsc{Composition}$$

which essentially states that if executing $S_1$ from any state satisfying $P$ terminates (if at all) in some state satisfying $R$, and executing $S_2$ from any state satisfying $R$ terminates (if at all) in some state satisfying $Q$, then it is the case that executing the composition $S_1; S_2$ from any state satisfying $P$ terminates (if at all) in some state satisfying $Q$. The proof system is sound and relatively complete w.r.t. the denotational semantics of the programming language (see, e.g., [23,19]).

*Hoare Logic Contracts.* One can view a Hoare triple $\{P\}S\{Q\}$ as a *contract* $C = (P, Q)$ imposed on the program $S$. In many contexts it is meaningful to separate the contract from the program; for instance, if the program is yet to be implemented. In our earlier work [12], we gave such contracts a denotational semantics as follows:

$$[\![C]\!] \stackrel{\mathsf{def}}{=} \{(s, s') \mid \forall \mathcal{I}.\ (s \models_{\mathcal{I}} P \Rightarrow s' \models_{\mathcal{I}} Q)\} \tag{2}$$

The rationale behind this definition is the following desirable property: a program *meets* a contract whenever its denotation is subsumed by the denotation of the contract, i.e., $S \models_{par} C$ if and only if $[\![S]\!] \subseteq [\![C]\!]$.

For example, for the contract $C_{odd}$ induced by (1) we have that $(s, s') \in [\![C_{odd}]\!]$ if and only if either $s(n) < 0$, or else $s'(r) = 0$ if $s(n)$ is even and $s'(r) = 1$ if $s(n)$ is odd. The denotation of $C_{even}$ is analogous.

*The Denotational Semantics of Programs with Procedure Contracts.* Let $S$ be a program with procedures, and let every declared procedure $p \in \mathcal{P}$ be equipped with a procedure contract $C_p$. *Procedure-modular verification* refers to techniques that verify every procedure in isolation. The key to this is to handle procedure calls by using the contract of the called procedure rather than its body (i.e., by *contracting* rather than by *inlining* [7]). In [12], a semantic justification of this is given by means of a *contract-relative* denotational semantics of statements. The intuition behind this semantics is that procedure calls are given a meaning through the denotation of the contract of the called procedure, rather than through the denotation of its body.

The contract-relative denotational semantics of a statement $S$, denoted $[\![S]\!]^{cr}$, is defined with the help of the *contract environment* $\rho_c$ that is induced by the procedure contracts, i.e., $\rho_c(p) \stackrel{\mathsf{def}}{=} [\![C_p]\!]$ for all $p \in \mathcal{P}$, as $[\![S]\!]^{cr} \stackrel{\mathsf{def}}{=} [\![S]\!]_{\rho_c}$. Notice that this definition does not involve solving any recursive equations (i.e., finding fixed points), and gives rise to a contract-relative notion of when a statement meets a contract, namely $S \models^{cr}_{par} C$ if and only if $[\![S]\!]^{cr} \subseteq [\![C]\!]$. This is exactly the correctness notion that is the target of procedure-modular verification. As shown in [12], this notion is *sound* w.r.t. the original notion $S \models_{par} C$, in the sense that $S \models^{cr}_{par} C$ entails $S \models_{par} C$. In other words, verifying a program procedure-modularly establishes that the program is correct w.r.t. its contract in the standard sense.

For example, the contract-relative semantics of $S_{even}$ is such that $(s, s') \in [\![S_{even}]\!]^{cr}$ if either $s(n) < 0$, or $s(n) = 0$ and $s' = s[r \mapsto 1]$, or else $s'(r) = 1$

if $s(n)$ is even and $s'(r) = 0$ if $s(n)$ is odd. The contract-relative semantics of $S_{odd}$ is analogous. Then, it is easy to check that both $S_{even} \models^{cr}_{par} C_{even}$ and $S_{odd} \models^{cr}_{par} C_{odd}$ hold.

## 4 An Abstract Contract Theory

This section presents an abstract contract theory for programs with procedures. The theory builds on the basic notion of *denotation* as a binary relation over states. As we will show later, it is both an abstraction of the denotational semantic view on programs with procedures and procedure contracts presented in Sections 3.1 and 3.2, and an instantiation of the meta-theory described in Section 2.2.

### 4.1 Components

In the context of a concrete programming language, we view a component as a module, consisting of a collection of procedures that are *provided* by the module. The module may call *required* procedures that are external to the module. The way the provided procedures transform the program state upon a call depends on how the required procedures transform the state. We take this observation as the basis of our abstract setting, in which state transformers are modelled as denotations (i.e., as binary relations over states). A component will thus be simply a mapping from denotations of the required procedures to denotations of the provided ones, both captured through the notion of procedure environments.

The contract theory is abstract, in that it is not defined for a particular programming language, and may be instantiated with any procedural language. As with the meta-theory, the abstract contract theory is also defined only on the semantic level.

Recall the notions and notation from Section 3.1. A component *interface* $I = (P^-, P^+)$ is a pair of disjoint, finite sets of procedure names, of the required and the provided ones, respectively.

**Definition 1 (Component).** *A* component $m$ *with interface* $I_m = (P^-_m, P^+_m)$ *is a mapping* $m : \mathbf{Env}_{P^-_m} \to \mathbf{Env}_{P^+_m}$.

Let $\mathcal{M}$ denote the universe of all components over $\mathcal{P}$.

We assume that any system is built up from a set of *base components*, the simplest components from which more complex components are then obtained by composition. The base components must be *monotonic* functions over the lattice defined in Section 3.1.

When $P^-_m = \varnothing$, we shall identify $m$ with an element of $\mathbf{Env}_{P^+_m}$. In other words, when a component is *closed*, i.e., is not dependent on any external procedures, the provided environment is constant.

**Definition 2 (Component composability).** *Two components* $m_1$ *and* $m_2$ *are* composable *iff* $P^+_{m_1} \cap P^+_{m_2} = \varnothing$.

When defining the composition of two components, particular care is required in the treatment of procedure names that are provided by one of the components while required by the other. Let $\mu x.\ f(x)$ denote the least fixed-point of a function $f$, when it exists.

**Definition 3 (Component composition).** *Given two composable components* $m_1 : \mathbf{Env}_{P^-_{m_1}} \rightarrow \mathbf{Env}_{P^+_{m_1}}$ *and* $m_2 : \mathbf{Env}_{P^-_{m_2}} \rightarrow \mathbf{Env}_{P^+_{m_2}}$*, their* composition *is defined as a mapping* $m_1 \times m_2 : \mathbf{Env}_{P^-_{m_1 \times m_2}} \rightarrow \mathbf{Env}_{P^+_{m_1 \times m_2}}$ *such that:*

$$P^+_{m_1 \times m_2} \stackrel{\mathsf{def}}{=} P^+_{m_1} \cup P^+_{m_2}$$

$$P^-_{m_1 \times m_2} \stackrel{\mathsf{def}}{=} (P^-_{m_1} \cup P^-_{m_2}) \setminus (P^+_{m_1} \cup P^+_{m_2})$$

$$m_1 \times m_2 \stackrel{\mathsf{def}}{=} \lambda \rho^-_{m_1 \times m_2} \in \mathbf{Env}_{P^-_{m_1 \times m_2}}.\ \mu\rho.\ \chi^+_{m_1 \times m_2}(\rho)$$

*where* $\chi^+_{m_1 \times m_2} : \mathbf{Env}_{P^+_{m_1 \times m_2}} \rightarrow \mathbf{Env}_{P^+_{m_1 \times m_2}}$ *is defined, in the context of a given* $\rho^-_{m_1 \times m_2} \in \mathbf{Env}_{P^-_{m_1 \times m_2}}$*, as follows. Let* $\rho^+_{m_1 \times m_2} \in \mathbf{Env}_{P^+_{m_1 \times m_2}}$*, and let* $\rho^-_{m_1} \in \mathbf{Env}_{P^-_{m_1}}$ *be the environment defined by:*

$$\rho^-_{m_1}(p) \stackrel{\mathsf{def}}{=} \begin{cases} \rho^+_{m_1 \times m_2}(p) & \textit{if } p \in P^-_{m_1} \cap P^+_{m_2} \\ \rho^-_{m_1 \times m_2}(p) & \textit{if } p \in P^-_{m_1} \setminus P^+_{m_2} \end{cases}$$

*and let* $\rho^-_{m_2} \in \mathbf{Env}_{P^-_{m_2}}$ *be defined symmetrically. We then define:*

$$\chi^+_{m_1 \times m_2}(\rho^+_{m_1 \times m_2})(p) \stackrel{\mathsf{def}}{=} \begin{cases} m_1(\rho^-_{m_1})(p) & \textit{if } p \in P^+_{m_1} \\ m_2(\rho^-_{m_2})(p) & \textit{if } p \in P^+_{m_2} \end{cases}$$

In the above definition, $\chi^+_{m_1 \times m_2}$ represents the denotations of the procedure *bodies* of the procedures provided by the two composed components, given denotations of procedure *calls* to the same procedures. The choice of least fixed-point will be crucial for the proof of Theorem 2(i) in Section 4.2 below.

The definition is well-defined, in the sense that the stated least fixed-points exist, and the resulting components are monotonic functions.

**Theorem 1.** *Component composition is well-defined.*

The existence of a least fixed-point follows from the Knaster-Tarski Fixed-Point Theorem, as stated, e.g., in [23]. It can then be shown, by structural induction, that composition is well-defined. For lack of space, the proofs of all theorems, some of which are conceptually not very involved but rather verbose, are omitted here. The full proofs can be found in the accompanying technical report [17].

### 4.2 Denotational Contracts

We now define the notion of denotational contracts $c$ in the style of *assume/guarantee contracts* [4,6]. Contracts shall also be given interfaces.

**Definition 4 (Denotational contract).** *A* denotational contract $c$ *with interface* $I_c = (P_c^-, P_c^+)$ *is a pair* $(\rho_c^-, \rho_c^+)$, *where* $\rho_c^- \in \mathbf{Env}_{P_c^-}$ *and* $\rho_c^+ \in \mathbf{Env}_{P_c^+}$.

The intended interpretation of the environment pair is as follows: *assuming* that the denotation of every called procedure $p \in P_c^-$ is subsumed by $\rho_c^-(p)$, then it is *guaranteed* that the denotation of every provided procedure $p' \in P_c^+$ is subsumed by $\rho_c^+(p')$.

**Definition 5 (Contract implementation).** *A component* $m$ *with interface* $I_m = (P_m^-, P_m^+)$ *is an* implementation *for, or* implements, *a contract* $c = (\rho_c^-, \rho_c^+)$ *with interface* $I_c = (P_c^-, P_c^+)$, *denoted* $m \models c$, *iff* $P_c^- \subseteq P_m^-$, $P_m^+ \subseteq P_c^+$, *and* $m(\rho_c^- \sqcup \rho^{\top}_{P_m^- \setminus P_c^-}) \sqsubseteq \rho_c^+$.

The reason for not requiring the interfaces to be equal is that we aim at a subset relation between components implementing a contract and those implementing a refinement of said contract, in the meta-theory instantiation.

For a mapping $h : A \to B$ and set $A' \subseteq A$, let $h_{|A'}$ denote as usual the restriction of $h$ on $A'$.

**Definition 6 (Contract environment).** *A component* $m$ *is an* environment *for contract* $c$ *iff, for any implementation* $m'$ *of* $c$, $m$ *and* $m'$ *are composable, and* $\forall \rho^-_{m \times m'} \in \mathbf{Env}_{P^-_{m \times m'}}.\ (m \times m')(\rho^-_{m \times m'})_{|P_c^+} \sqsubseteq \rho_c^+$.

Intuitively, an environment of a contract $c$ is then a component such that when it is composed with an implementation of $c$, the composition will operate satisfactorily with respect to the guarantee of the contract.

We will now define the refinement relation, and the conjunction and composition operations, on contracts.

**Definition 7 (Contract refinement).** *A contract* $c$ refines *contract* $c'$, *denoted* $c \preceq c'$, *iff* $\rho_{c'}^- \sqsubseteq \rho_c^-$ *and* $\rho_c^+ \sqsubseteq \rho_{c'}^+$, *where* $\sqsubseteq$ *is the partial order relation defined in Section 3.1.*

The refinement relation reflects the intention that if a contract $c$ refines another contract $c'$, then any component implementing $c$ should also implement $c'$.

**Definition 8 (Contract conjunction).** *The* conjunction *of two contracts* $c_1 = (\rho_{c_1}^-, \rho_{c_1}^+)$ *and* $c_2 = (\rho_{c_2}^-, \rho_{c_2}^+)$ *is the contract* $c_1 \wedge c_2 \stackrel{\mathsf{def}}{=} (\rho_{c_1}^- \sqcup \rho_{c_2}^-, \rho_{c_1}^+ \sqcap \rho_{c_2}^+)$, *where* $\sqcup$ *and* $\sqcap$ *are the* lub *and* glb *operations of the lattice, respectively.*

This definition is consistent with the intention that any contract that refines $c_1 \wedge c_2$ should also refine $c_1$ and $c_2$ individually. The interface of $c_1 \wedge c_2$ is then $I_{c_1 \wedge c_2} = (P_{c_1}^- \cup P_{c_2}^-, P_{c_1}^+ \cap P_{c_2}^+)$. Note that while this is the interface in general, conjunction of contracts is typically used to merge different viewpoints of *the same* component, and in that case $I_{c_1} = I_{c_2} = I_{c_1 \wedge c_2}$.

**Definition 9 (Contract composability).** *Two contracts* $c_1 = (\rho_{c_1}^-, \rho_{c_1}^+)$ *and* $c_2 = (\rho_{c_2}^-, \rho_{c_2}^+)$ *with interfaces* $I_{c_1} = (P_{c_1}^-, P_{c_1}^+)$ *and* $I_{c_2} = (P_{c_2}^-, P_{c_2}^+)$ *are* composable *if: (i)* $P_{c_1}^+ \cap P_{c_2}^+ = \varnothing$, *(ii)* $\forall p \in P_{c_1}^- \cap P_{c_2}^+.\ \rho_{c_2}^+(p) \subseteq \rho_{c_1}^-(p)$, *and (iii)* $\forall p \in P_{c_2}^- \cap P_{c_1}^+.\ \rho_{c_1}^+(p) \subseteq \rho_{c_2}^-(p)$.

The conditions for composability ensure that the mutual guarantees of the two contracts meet each other's assumptions.

**Definition 10 (Contract composition).** *The* composition *of two composable contracts* $c_1 = (\rho^-_{c_1}, \rho^+_{c_1})$ *and* $c_2 = (\rho^-_{c_2}, \rho^+_{c_2})$*, with interfaces* $I_{c_1} = (P^-_{c_1}, P^+_{c_1})$ *and* $I_{c_2} = (P^-_{c_2}, P^+_{c_2})$*, respectively, is the contract* $c_1 \otimes c_2 \stackrel{\mathsf{def}}{=} (\rho^-_{c_1 \otimes c_2}, \rho^+_{c_1} \sqcup \rho^+_{c_2})$*, where:*

$$\rho^-_{c_1 \otimes c_2} \stackrel{\mathsf{def}}{=} (\rho^-_{c_1} \sqcap \rho^-_{c_2})\big|_{(P^-_{c_1} \cup P^-_{c_2}) \setminus (P^+_{c_1} \cup P^+_{c_2})}$$

The interface of $c_1 \otimes c_2$ is $I_{c_1 \otimes c_2} = ((P^-_{c_1} \cup P^-_{c_2}) \setminus (P^+_{c_1} \cup P^+_{c_2}), P^+_{c_1} \cup P^+_{c_2})$.

**Theorem 2.** *For any composable contracts* $c_1$ *and* $c_2$*, and any implementations* $m_1 \models c_1$ *and* $m_2 \models c_2$*,* $m_1$ *and* $m_2$ *are composable, and* $c_1 \otimes c_2$ *is the least contract (w.r.t. refinement order) for which the following properties hold:*

*(i)* $m_1 \times m_2 \models c_1 \otimes c_2$,
*(ii) if* $m$ *is an environment to* $c_1 \otimes c_2$*, then* $m_1 \times m$ *is an environment to* $c_2$*,*
*(iii) if* $m$ *is an environment to* $c_1 \otimes c_2$*, then* $m \times m_2$ *is an environment to* $c_1$*.*

## 5 Connection to Meta-Theory

In this section we show that the abstract contract theory presented in Section 4 instantiates the meta-theory described in Section 2.2.

In our instantiation of the meta-theory, we consider as the abstract component universe $\mathbb{M}$ the same universe of components $\mathcal{M}$ as defined in Section 4.1. To distinguish the contracts of the meta-theory from those of the abstract theory, we shall always denote the former by $\mathcal{C}$ and the latter by $c$. Recall that a contract $\mathcal{C}$ is a pair $(E, M)$, where $E, M \subseteq \mathcal{M}$. The formal connection between the two notions is established with the following definition.

**Definition 11 (Induced contract).** *Let* $c$ *be a denotational contract. It* induces *the contract* $\mathcal{C}_c = (E_c, M_c)$*, where* $E_c \stackrel{\mathsf{def}}{=} \{m \in \mathcal{M} \mid m \text{ is an environment for } c\}$ *and* $M_c \stackrel{\mathsf{def}}{=} \{m \in \mathcal{M} \mid m \models c\}$*.*

Since contract implementation requires that the implementing component's provided functions are a subset of the contract's provided functions, every component $m$ such that $P^+_m \cap P^+_c = \varnothing$ is composable with every component in $M_c$.

The definitions of implementation, refinement and conjunction of denotational contracts make this straightforward definition of induced contracts possible, so that it directly results in refinement as set membership and conjunction as lub w.r.t. the refinement order.

**Theorem 3.** *The contract theory of Section 4 instantiates the meta-theory of Benveniste et al. [5], in the sense that composition of components is associative and commutative, and for any two contracts* $c_1$ *and* $c_2$*:*

*(i)* $c_1 \preceq c_2$ *iff* $\mathcal{C}_{c_1}$ *refines* $\mathcal{C}_{c_2}$ *according to the definition of the meta-theory,*

*(ii)* $\mathcal{C}_{c_1 \wedge c_2}$ *is the conjunction of* $\mathcal{C}_{c_1}$ *and* $\mathcal{C}_{c_2}$ *as defined in the meta-theory, and*
*(iii)* $\mathcal{C}_{c_1 \otimes c_2}$ *is the composition of* $\mathcal{C}_{c_1}$ *and* $\mathcal{C}_{c_2}$ *as defined in the meta-theory.*

The proof is straightforward, since many definitions of the contract theory are deliberately similar to their counterparts in the meta-theory.

Let us now return to our example from Section 3. When applying Contract Based Design, contracts at the more abstract level will be decomposed into contracts at the more concrete level. So, for our example, we might have at the top level a contract $c = (\rho_c^-, \rho_c^+)$ with interface $(\varnothing, \{even, odd\})$, where $\rho_c^- = \varnothing$, and where $\rho_c^+ \in \mathbf{Env}_{P_c^+}$ maps *even* to the set of pairs $(s, s')$ such that whenever $s(n)$ is non-negative and even, then $s'(r) = 1$, and when $s(n)$ is non-negative and odd, then $s'(r) = 0$, and maps *odd* in a dual manner. This contract could then be decomposed into two contracts $c_{even}$ and $c_{odd}$, so that $\rho^+_{c_{even}}(even) \overset{\mathsf{def}}{=} \rho_c^+(even)$ and $\rho^-_{c_{even}}(odd) \overset{\mathsf{def}}{=} \rho_c^+(odd)$, and $c_{odd}$ is analogous. Then, we would have $c_{even} \otimes c_{odd} \preceq c$, and for any two components $m_{even}$ and $m_{odd}$ such that $m_{even} \models c_{even}$ and $m_{odd} \models c_{odd}$, it would hold that $m_{even} \times m_{odd} \models c$.

## 6 Connection to Programs with Procedures

In this section we discuss how our abstract contract theory from Section 4 relates to programs with procedures as presented in Section 3.1, and how it relates to Hoare logic and procedure-modular verification as presented in Section 3.2.

First, we define how to abstract the denotational notion of procedures into components in the abstract theory, based on the function $\xi$ from Section 3.1.

**Definition 12 (From procedure sets to components).** *For any set of procedures* $P^+$*, calling procedures* $P'$*, we define the component* $m : \mathbf{Env}_{P_m^-} \to \mathbf{Env}_{P_m^+}$*, where* $P_m^- \overset{\mathsf{def}}{=} P' \setminus P_m^+$ *and* $P_m^+ \overset{\mathsf{def}}{=} P^+$*, so that* $\forall \rho_m^- \in \mathbf{Env}_{P_m^-}.\ \forall p \in P_m^+.\ m(\rho_m^-)(p) \overset{\mathsf{def}}{=} [\![S_p]\!]_{\rho_m^-}$.

As the next result shows, procedure set abstraction and component composition commute. Together with commutativity and associativity of component composition, this means that the initial grouping of procedures into components is irrelevant, and that one can start with abstracting each individual procedure into a component.

**Theorem 4.** *For any two disjoint sets of procedures* $P_1^+$ *and* $P_2^+$*, abstracted individually into components* $m_1$ *and* $m_2$*, respectively, and* $P_1^+ \cup P_2^+$ *abstracted into component* $m$*, it holds that* $m_1 \times m_2 = m$.

The result is a direct consequence of Definition 12, Definition 3, and the well-known Bekić's Lemma [3] about simultaneous fixed-points.

*Component abstraction example.* Let us illustrate the theorem on our even-odd example (however, the example does not really illustrate Bekić's Lemma, since the two procedures do not call themselves).

By Definition 12, the procedure set $\{even\}$ is abstracted into component $m_{even} : \mathbf{Env}_{\{odd\}} \rightarrow \mathbf{Env}_{\{even\}}$ with interface $(\{odd\}, \{even\})$, so that $\forall \rho^- \in \mathbf{Env}_{\{odd\}}.\ m(\rho^-)(even) = [\![S_{even}]\!]_{\rho^-}$. By definition, $[\![S_{even}]\!]_{\rho^-}$ is equal to $[\![S_{even}]\!]^{\rho_0^+}_{\rho^-}$, where $\rho_0^+$ is the least fixed point of $\xi : \mathbf{Env}_{\{even\}} \rightarrow \mathbf{Env}_{\{even\}}$ defined by $\xi(\rho^+)(even) \stackrel{\mathsf{def}}{=} [\![S_{even}]\!]^{\rho^+}_{\rho^-}$ for any $\rho^+ \in \mathbf{Env}_{\{even\}}$. Notice, however, that procedure *even* does not have any calls to itself, so $[\![S_{even}]\!]^{\rho_0^+}_{\rho^-}$ does not really depend on $\rho^+$. Then, for any $\rho^- \in \mathbf{Env}_{\{odd\}}$, $(s, s') \in m(\rho^-)(even)$ if either $s(n) = 0$ and $s' = s[r \mapsto 1]$, or else if $s(n) > 0$ and $(s[n \mapsto s(n)-1], s') \in \rho^-(odd)$.

Similarly, the procedure set $\{odd\}$ is abstracted into component $m_{odd} : \mathbf{Env}_{\{even\}} \rightarrow \mathbf{Env}_{\{odd\}}$ with interface $(\{even\}, \{odd\})$, so that $\forall \rho^- \in \mathbf{Env}_{\{even\}}.\ m(\rho^-)(odd) = [\![S_{odd}]\!]_{\rho^-}$. Then, for any $\rho^- \in \mathbf{Env}_{\{even\}}$, $(s, s') \in m(\rho^-)(odd)$ if either $s(n) = 0$ and $s' = s[r \mapsto 0]$, or else if $s(n) > 0$ and $(s[n \mapsto s(n) - 1], s') \in \rho^-(even)$.

Now, applying Definition 12 to the whole (closed) program yields a component $m : \mathbf{Env}_{\varnothing} \rightarrow \mathbf{Env}_{\{even,odd\}}$ with interface $(\varnothing, \{even, odd\})$, so that $\forall \rho^- \in \mathbf{Env}_{\varnothing}.\ \forall p \in \{even, odd\}\ .\ m(\rho^-)(p) = [\![S_p]\!]_{\rho^-}$. Recall the denotations $[\![S_{even}]\!]_{\rho^-}$ and $[\![S_{odd}]\!]_{\rho^-}$ from the end of Section 3.1.

Components $m_{even}$ and $m_{odd}$ are composable, and by Definition 3, their composition has (the same) interface $(\varnothing, \{even, odd\})$, and is (also) a mapping $m_{even} \times m_{odd} : \mathbf{Env}_{\varnothing} \rightarrow \mathbf{Env}_{\{even,odd\}}$.

Finally, note that function $\chi^+_{m_{even} \times m_{odd}} : \mathbf{Env}_{\{even,odd\}} \rightarrow \mathbf{Env}_{\{even,odd\}}$ is exactly the function $\xi$ in the context of the interface $(\varnothing, \{even, odd\})$. This can be seen by first noting that since $\mathbf{Env}_{\varnothing} = \varnothing$, we have that $\chi^+_{m_{even} \times m_{odd}}$ only depends on its arguments. Furthermore, for all $\rho^+ \in \mathbf{Env}_{\{even,odd\}}$, if $\rho^+_{odd} \stackrel{\mathsf{def}}{=} \rho^+\big|_{\{odd\}}$ and $\rho^+_{even} \stackrel{\mathsf{def}}{=} \rho^+\big|_{\{even\}}$ we have that, since $odd \in P^-_{even} \cap P^+_{odd}$, then $\chi^+_{m_{even} \times m_{odd}}(\rho^+)(even) = m_{even}(\rho^+_{odd})(even) = [\![S_{even}]\!]_{\rho^+_{odd}} = [\![S_{even}]\!]^{\rho^+} = \xi(\rho^+)(even)$. Similarly $\chi^+_{m_{even} \times m_{odd}}(\rho^+)(odd) = \xi(\rho^+)(odd)$. We therefore have $m_{even} \times m_{odd} = m$.

We now define how to abstract Hoare logic contracts into denotational contracts, in terms of the contract environment $\rho_c$ defined in Section 3.2.

**Definition 13 (From Hoare logic contracts to denotational contracts).** *For a procedure $p$ with Hoare logic contract $C_p$, calling other procedures $P^-$, we define the denotational contract $c_p = (\rho^-_{c_p}, \rho^+_{c_p})$ with interface $P^+_{c_p} \stackrel{\mathsf{def}}{=} \{p\}$ and $P^-_{c_p} \stackrel{\mathsf{def}}{=} P^-$, so that $\rho^+_{c_p}(p) \stackrel{\mathsf{def}}{=} \rho_c(p)$, and $\forall p' \in P^-.\ \rho^-_{c_p}(p') = \rho_c(p')$.*

In this way, conceptually, denotational contracts become assume/guarantee-style specifications over Hoare logic procedure contracts: assuming that all (external) procedures called by a procedure $p$ transform the state according to their Hoare logic contracts, procedure $p$ obliges itself to do so as well.

We now show that if a procedure implements a Hoare logic contract, then the abstracted component will implement the abstracted contract, and vice versa. Together with Theorem 4, this result allows the *procedure-modular verification* of abstract components.

**Theorem 5.** *For any procedure $p$ with procedure contract $C_p$, abstracted into component $m_p$ with contract $c_p$, we have $S_p \models^{cr}_{par} C_p$ iff $m_p \models c_p$.*

The result follows mainly from Definitions 12 and 13, and the denotational semantics given in Section 3.

Returning to the example from Sections 3 and 5, we can abstract the procedure set $\{even\}$ into component $m_{even}$, with interface $(\{odd\}, \{even\})$, which would be a function $\mathbf{Env}_{\{odd\}} \to \mathbf{Env}_{\{even\}}$, and $\forall \rho^- \in \mathbf{Env}_{\{odd\}}.\ m(\rho^-)(even) = [\![S_{even}]\!]_{\rho^-}$. The denotational contracts $c_{even}$ and $c_{odd}$ resulting from the decomposition shown in Section 5, would be exactly the abstraction of the Hoare Logic contracts $C_{even}$ and $C_{odd}$ shown in Section 3.2. They would both be part of the contract environment used in procedure-modular verification, for example when verifying that $S_{even} \models^{cr}_{par} C_{even}$, which would entail $m_{even} \models c_{even}$. Thus, by applying standard procedure-modular verification at the source code level, we prove the top-level contract $c$ proposed in Section 5.

## 7 Conclusion

We presented an abstract contract theory for procedural languages, based on denotational semantics. The theory is shown to be an instance of the meta-theory of [5], and at the same time an abstraction of the standard denotational semantics of procedural languages. We believe that our contract theory can be used to support the development of cyber-physical and embedded systems by the design methodology supported by the meta-theory, allowing the individual procedures of the embedded software to be treated as any other system component. The work also strengthens the claims of the meta-theory of distilling the notion of contracts to its essence, by showing that it is applicable also in the context of procedural programs and deductive verification. Finally, this work serves as a preparation for combining our contract theory for procedural programs with other instantiations of the meta-theory. In future work we plan to investigate the utility of our contract theory on real embedded systems taken from the automotive industry, where not all components are procedural programs, or even software (cf. our previous work, e.g., [11]). We also plan to extend our toy imperative language with additional features, such as procedure parameters and return values. Furthermore, we plan to extend the contract theory to capture program traces by developing a finite-trace semantics, to enable its use in the specification and verification of temporal properties. Lastly, we plan to combine our contract theory with an existing contract theory for hybrid systems [20].
